# The role of communication in the operating theatre: attitudes of anaesthesiology trainees

**DOI:** 10.1007/s11845-026-04442-8

**Published:** 2026-05-15

**Authors:** Genevieve L. Belanger, Conall Fitzgerald, Sinéad O’Shaughnessy

**Affiliations:** 1 School of Medicine, University of Dublin Trinity College, 2 College Green, Dublin 2, Ireland; 2 Department of Otolaryngology, Head and Neck Surgery, St. James’s Hospital, Dublin 8, Ireland; 3 Department of Anaesthesia and Intensive Care Medicine, Mater Misericordiae University Hospital, Dublin 7, Ireland

**Keywords:** Anaesthesiology trainees, Communication skills, Operating theatre, Patient safety

## Abstract

**Introduction:**

Effective teamwork in the operating theatre is essential for safety and optimal patient outcomes. Extensive research has examined this topic from the perspectives of anaesthesiology consultant and surgeons; however, limited attention has been given to anaesthesiology trainees. Hence, this study explores anaesthesiology trainees’ attitudes towards communication and leadership in the operating theatre.

**Methods:**

A questionnaire-based study was conducted among anaesthesiology trainees in Irish hospitals. The questionnaire was adapted from the Operating Room Management Attitudes Questionnaire (OMRAQ). Data collected included demographics, trainee attitudes toward communication and leadership, perceptions of team dynamics and communication-related errors. Responses were analysed through descriptive statistical analysis.

**Results:**

A total of 74 trainees responded. The majority expressed a strong preference for collaborative leadership and open communication, with 95% of trainees supporting junior staff participation in decision making. However, 69% reported a lack of pre-operative briefings and 84% stated that post-operative debriefings were uncommon, demonstrating pitfalls in theatre communication. Furthermore, 85% acknowledged making errors due to poor communication, with over half reporting witnessing such errors from other members of theatre. Trainees supported the need for confidential error-reporting systems but expressed hesitation about openly reporting incidents.

**Conclusion:**

Anaesthesiology trainees value teamwork and communication but perceive significant gaps between best practices and current hospital procedures. These findings highlight the need for systemic interventions, including structured communication training, leadership development, and the promotion of psychological safety within theatre. Addressing these areas could improve communication dynamics, reduce medical errors, and enhance patient safety.

## Introduction

Current literature emphasises the role of high-quality communication in the operating theatre. Effective communication and teamwork among all members of the surgical team have been shown to reduce medical errors and enhance patient management [1–3]. While extensive research has demonstrated the attitudes and impact of surgeons and surgical trainees, far less attention has been given to anaesthetists, particularly anaesthesiology trainees, despite their critical role in the operating theatre.

Many studies have highlighted the importance of the anaesthetist in effective operating theatre communication, with failures in the area leading to patient errors [1–4]. Increasingly, there is recognition of the need for enhanced communication training across the speciality to improve overall teamwork and efficiency in the operating theatre [5, 6].

Anaesthesiology trainees face distinct challenges compared with senior staff when developing effective communication. While interventions designed to improve communication skills among trainees have been introduced, there remains stigma and resistance from senior staff and other members of the operating theatre [4]. A systematic review and meta-analysis on the topic emphasises the importance of fostering an open and supportive operating theatre environment, where trainees feel safe to express their views and concerns [7]. A recognised and challenging barrier is the ‘hidden hierarchy’. This describes a long-held and persistent culture of valuing communication differently depending on the status and seniority of the team member. Addressing and dismantling such hierarchies is important to enhance both trainee communication and overall patient safety [8].

Effective communication is required not only between senior operating team members, but also between senior staff and trainees. Strong communication practices help to alleviate trainees’ anxiety, worry, and fear when entering the operating theatre [9]. This study aims to assess the attitudes of anaesthesiology trainees in Irish hospitals regarding communication within the operating theatre. This research hopes to inform future initiatives by providing recommendations for improving communication training and fostering a more inclusive and effective operating theatre environment.

## Methods

### Study design

One of the most widely used questionnaires for assessing attitudes among various operating theatre team members within Europe is the Operating Room Management Attitudes Questionnaire (ORMAQ) [10]. The ORMAQ questionnaire was adapted for use within the Irish anaesthesia trainee programme for this study.

The questionnaire details initial demographic data, including respondent’s sex (male/female), years of anaesthesia practice, type of medical degree (undergraduate/postgraduate), and any prior medical speciality training.

The body of the questionnaire was divided into four sections. The first section focused on key themes such as leadership structure, confidence in asserting oneself, information sharing, teamwork, work values and organisational climate, as categorised by Flin in their 2003 study on anaesthetists’ attitudes in the operating theatre. Respondents were asked to indicate the extent to which they agreed with each statement using a five-point Likert scale.

The second section assessed the quality of communication between anaesthesiology trainees and other operating theatre members, including anaesthesiology consultants, trainees’, and nurses, consultant and trainee surgeons, operating theatre managers and other nursing staff. Respondents rated communication quality on a five-category scale according to the original ORMAQ format from very low to very high. Respondents were asked to evaluate five statements related to errors within the operating theatre, on a five-point Likert scale.

Finally, trainee leadership style was evaluated using the ORMAQ (autocratic, consultative, joint and delegative) as well as trainees’ observations of the different leadership styles of surgeons, fellow anaesthetists and nurses.

### Procedure

Questionnaires were delivered to anaesthesiology trainees at the end of a communication skills workshop distributed in a peer-teaching fashion. The workshop was led by an anaesthetic trainee with a higher degree in medical education and was mandatory, as per the College of Anaesthesiologists of Ireland. Thus, it captured comprehensive information on trainees’ communication experiences in all hospitals in Ireland participating in anaesthesia training. All questionnaires were completed anonymously. The only descriptive data related to the individual participants was their sex (male or female). Microsoft Microsoft Excel was used to conduct data analysis, with each questionnaire assigned a randomised ID number for analytical purposes.

## Results

### Demographics

The survey captured responses from a total of 74 anaesthesiology trainees across Irish teaching hospitals (Table 1). Of the total participants, 43 identified as male (58%) and 31 as female (42%). Most participants had less than one year of anaesthesia training (31 participants), while 8 had completed one year, 16 had one and a half years of training, 3 had two and a half years, 4 had three years and 1 participant had more than three years of training. The majority (46 [61%]) of anaesthesiology trainees completed an undergraduate medical degree, versus 28 who completed postgraduate medical degrees (39%). Twenty-three (31%) participants reported prior medical speciality training with 7 having experience in general medicine, 5 in surgical training, 5 in emergency medicine and 2 in nursing. Other specialities represented included psychiatry, orthopaedics, paediatrics, pharmacy and radiography. A detailed breakdown of participant demographics and prior specialty training is provided.

**Table 1:** Demographics of study respondents

Variable	Category	Number of participants	Percentage of total participants (%)
Sex	Male	43	58%
	Female	31	42%
Years of Anaesthesia Training	< 1 year	31	
	1 year	8	
	1.5 years	16	
	2 years	11	
	2.5 years	3	
	3 years	4	
	3.5 years	1	
Type of Medical Degree	Undergraduate	46	61%
	Postgraduate	28	39%
Other Medical Specialty Training	General Internal Medicine	7	
	General Surgery	5	
	Emergency Department	5	
	Nursing	2	
	Orthopaedics	1	
	Paediatrics	1	
	Pharmacy	1	
	Radiography	1	

### Teamwork and collaboration

Most anaesthesiology trainees expressed a strong preference for a collaborative team environment in the operating theatre. Notably, 76% of participants strongly agreed that senior staff should encourage questions from junior medical and nursing staff throughout operations where appropriate (Table 2). Similarly, most anaesthesiology trainees valued teamwork with 94% agreeing or strongly agreeing that they enjoy being a part of a team-based environment.

**Table 2:** ORMAQ responses for teamwork and communication

**Likert Scale Ratings**	**Q2: Senior staff should encourage questions from junior medical and nursing staff during operations if appropriate**	**Q6: Doctors who encourage suggestions from operating theatre team members are weak leaders**	**Q8: A regular post-operative debriefing of procedures is an important part of developing effective team co-coordination**	**Q9: Regular debriefing takes place at my hospital**	**Q10A: Team members in charge should verbalise plans for procedures or actions and should be sure that the information is understood**	**Q10B: The previous (10a) happens most of the time at my hospital**	**Q11: Junior operating theatre team should not question decisions made by senior personnel**	**Q13: The only people qualified to give me feedback are members of my own profession**
Strongly disagree (1)	0	74.3	4.1	40.5	0	9.5	52.8	39.2
Disagree (2)	0	18.9	1.4	43.2	0	39.2	37.9	48.6
Neutral (3)	1.4	2.7	12.2	12.2	4.1	19.0	9.5	5.4
Agree (4)	22.9	1.4	58.1	1.6	39.1	31.1	0	5.4
Strongly agree (5)	75.7	2.7	24.3	2.7	56.6	1.4	0	0
**Likert Scale Ratings**	**Q14: It is better to agree with other operating theatre team members than to voice a different opinion**	**Q15A: The pre session team briefing is important for safety and effective team management**	**Q15B: A pre session briefing takes place most of the time at my hospital**	**Q19: I enjoy working as part of a team**	**Q20: If I perceive a problem with the management of a patient, I will speak up, regardless of who might be affected**			
Strongly disagree (1)	18.9	1.4	27.0	0	1.4			
Disagree (2)	66.2	1.4	42.0	2.7	11.0			
Neutral (3)	9.5	12.2	14.9	4.1	27.4			
Agree (4)	5.5	55.4	13.5	44.6	52.1			
Strongly agree (5)	0	29.7	2.7	48.7	8.2			

Few trainees felt that they performed their best work alone; however, most recognised the value of other team members’ input. Seventy-four per cent disagreed that “doctors who encourage suggestions from operating theatre members are weak leaders.” Furthermore, this was not limited to the speciality of anaesthesia with 88% disagreeing or strongly disagreeing that external professionals cannot provide useful comments or feedback.

Despite a clear preference for collaboration and open communication, trainees identified many shortcomings in current operating theatre communication, particularly between senior and junior staff. Despite 96% of trainees either agreeing (39%) or strongly agreeing (57%) that senior team members should verbalise procedural plans to ensure full team understanding before operating, nearly half of respondents (49%) indicated that such conversations do not regularly occur in their hospital. Additionally, 85% of participants either agreed (55%) or strongly agreed (30%) that pre-session team briefings are crucial for effective team management and collaboration. However, 69% of participants stated that these briefings do not take place at their hospital. Similarly, 84% of participant’s either disagreed (43%) or strongly disagreed (41%) that regular post-operative debriefings take place despite high perception of their importance (83% agreement or strong agreement).

Regarding challenges to other team members, most trainees felt comfortable questioning senior staff with 91% disagreeing or strongly disagreeing that junior team members should not challenge senior members’ decisions. Eighty-five per cent of trainees disagreed with the statement that it is better to agree with other operating theatre members rather than voice their own opinion. However, despite this confidence to challenge, 68% of trainees expressed some level of concern about asking senior team members to take corrective action when needed. Descriptive statistics for all questions regarding teamwork and communication quality is included in the appendix 1.

### Communication quality and leadership styles by profession

When assessing quality of communication by profession, anaesthesiology consultants were rated the highest with 82% of trainees noting high quality or very high quality of communication of communication for this group (Table 3). Fellow anaesthesiology trainees were perceived as having stronger communication skills, with 50% being ranked as high quality communicators. Trainee surgeons fared slightly better than consultant surgeons with 53% ranking communication skills as either low or adequate for their trainee colleagues compared with for 70% consultants.

**Table 3:** ORMAQ responses for quality of communication by profession

Likert Scale Ratings	Consultant Surgeons	Anaesthesiology Consultants	Trainee Surgeons	Anaesthesiology Trainees	Theatre manager	Scrub/circulating nurse	Anaesthetic nurse
Strongly disagree (0)	1.4	0	4.1	0	6.8	4.1	0
Disagree (1)	27.0	4.1	16.2	2.7	13.5	21.6	5.5
Neutral (2)	43.2	13.6	36.9	9.5	32.4	36.5	16.4
Agree (3)	27.0	51.4	39.1	50.0	39.2	31.1	56.2
Strongly agree (4)	1.4	31.1	4.1	37.9	8.1	6.8	22.0

Regarding nursing staff, anaesthetic nurses were viewed most favourably with 56% of respondents rating their quality of communication as high. This was slightly lower for theatre managers (53%) and other nursing staff (38%). There was a recognition that communication quality varies widely between individuals, although the above ratings were based on group-level perceptions communication skill quality ratings above were made by group.

Trainees appreciated the value of senior leadership in high-stakes situations, with 78% agreeing or strongly agreeing that senior staff should take the lead during emergencies. However, it was not felt that leadership should be exclusive to senior members, as 95% disagreed or strongly disagreed that there are no circumstances where junior members should lead patient management.

Among leadership styles assessed in the questionnaire, almost half of trainees (49%) identified their own leadership style as joint decision making, in which leaders consult team members before making final decisions. This mirrored perceived leadership styles of (49% joint). Conversely, most trainees observed an autocratic leadership style from surgeons, where leaders make decisions independently and expect loyalty and obedience. Trainees’ opinions of nursing leadership style were more varied with 17% identifying an autocratic style, 16% a consultative style, 20% a joint style and 23% a delegative or passive style.

### Errors in communication

Most trainees (55%) had witnessed an error due to communication failure in the operating theatre (Table 4). Additionally, 85% of anaesthesiology trainees agreed or strongly agreed that they themselves had made errors in the operating theatre due to a lack of communication. Almost all trainees rejected the notion that communication errors are insignificant if the patient remains stable, with 92% disagreement or strongly disagreeing.

**Table 4:** ORMAQ responses for errors in communication

Likert Scale Ratings	Q1: I rarely witness an error where one or more team members has failed to communicate	Q2: I have made errors in theatre due to lack of communication	Q3: Errors made during patient management are not important as long as the patient improves	Q4: Medical errors are discussed to prevent recurrence	Q5: A confidential reporting system that documents medical errors is important for safety
Strongly disagree (0)	6.8	0	37.9	0	0
Disagree (1)	55.4	8.1	54.1	21.6	0
Neutral (2)	25.7	6.8	6.8	17.6	1.4
Agree (3)	12.2	70.3	1.4	46.0	52.7
Strongly agree (4)	0	14.9	0	14.9	46.0

Participants expressed ambivalence, however, regarding how often medical errors caused by poor communication are discussed to prevent recurrence with 46% remaining neutral. Only 36% of respondents agreed that they are encouraged by their leaders to report incidents they observe in the operating theatre as opposed to 32% who disagreed.

Nonetheless, there was overwhelming consensus on the importance of a confidential reporting system for documenting medical errors, with 99% of respondents either agreeing (53%) or strongly agreeing (46%) that such a system is crucial for patient safety.

## Discussion

This study indicates that anaesthesiology trainees strongly value teamwork and a collaborative environment among all operating theatre members. Notable discrepancies exist, however, between trainees’ perceptions of effective communication and actual hospital practices, particularly in areas such as pre-operative planning and debriefing. These findings are consistent with existing studies on the views of the anaesthesia team of appropriate hospital communication across various cultural settings [11–14].

Our study aligns with current literature in showing that trainee anaesthetists have a strong preference for a team-based approach to communication, reflected in results demonstrating high agreement with collaborative approaches both within and between different theatre staffing groups and prefers joint leadership styles between consultants and trainees. The current literature stresses how suboptimal teamwork can directly compromise patient safety. Current recommendations include closed-loop communication, silencing of mobile devices and overall situational awareness as key interventions to enhance teamwork and potentially improve surgical outcomes [15, 16]. Looking ahead, artificial intelligence may play a role in enhancing communication, accountability and teamwork [17].

One of the most striking findings of our study is the perception that surgeons have a lower overall communication quality compared with other operating theatre members. The presence of hierarchical structures within the theatre environment, often referred to as the ‘hidden hierarchy’, has a significantly detrimental effect on junior staff’s willingness to voice concerns unless actively encouraged by senior members [7, 18]. Power dynamics often dictate that individuals at similar levels (e.g. anaesthesiology consultants and consultant surgeons) are more likely to engage in direct communication, whereas junior staff and nurses may feel hesitant to challenge senior members [7]. Specifically for anaesthesiology trainees, fear and intimidation from senior staff has led to a reluctance to challenge the hidden hierarchy [18]. Novel medical education initiatives, particularly peer-led techniques may offer some solutions to shift this culture. Simulation-based training, where peers are encouraged to challenge erroneous decisions made by authority figures within a psychologically safe environment, represents a promising development [19]. Further recognition of and challenge to the “hidden hierarchy” within guidelines and medical curricula as well as early peer-led education initiatives may alleviate future trainees’ fear and foster greater leadership in operating theatre communication [20].

Anaesthesiology trainees regarded a joint leadership style as the most consistently used across all grades within their own speciality. This contrasted with their colleagues in surgery, who were perceived as using an autocratic style, particularly among consultants. Interestingly, these results mirror historical accounts of surgeon behaviour, rather than recent shifts in surgical leadership towards more transactional and mutual respect based models [21–23]. Significant cultural change takes time and requires a consistent, and dedicated approach. These results highlight an opportunity for enhanced communication and leadership training among surgeons within Irish hospitals to align with evolving leadership models and foster improved collaboration.

Failures in communication have long been identified as key contributors to medical errors [24], Over half of Irish anaesthesiology trainees reported witnessing a medical error due to communication failure, and 85% acknowledged having made errors themselves due to poor communication. Despite this awareness, trainees were divided on the question of whether to report errors non-anonymously, a significant concern, as reporting culture is essential for patient safety. The current literature demonstrates that anonymous and non-punitive error reporting systems significantly increase the reporting of errors and near misses within the operating theatre [25]. Notably, studies suggest that anaesthesiology trainees are more likely to report medical errors when under less direct supervision and when in a non-judgemental and open environment [26].

Nearly all trainees in this study supported the implementation of a confidential medical error reporting system, recognising its value in improving patient safety. However, challenges remain, including underutilisation, fear of repercussions and a lack of integration into broader hospital culture [27, 28]. For such systems to be effective, they must be actively maintained, normalised, and supported by senior leadership as part of a wider hospital safety strategy.

The results of this study carry significant clinical implications for communication in the operating theatre. Many findings suggest that structured communication and teamwork interventions could meaningfully enhance the communication environment for trainees. These may include communication checklists, team training, and regular post-operative debriefings [29]. To overcome barriers such as limited accessibility and staff buy-in, these interventions must be supported by senior leadership and adopted by all operating team members [30].

The value of peers’ influence in driving cultural change in communication culture change should not be underestimated. Peer-to-peer communication was highly rated in this study. Peer-led communication skill initiatives offer considerable potential, with evidence showing they can be equally or even more effective than professional workshops [31, 32]. Peer teaching workshops have demonstrated improvements in teamwork and interdisciplinary communication skills and garnered higher satisfaction ratings than didactic methods [31, 32].

Another critical factor in fostering a culture of open communication is ensuring psychological safety when voicing concerns and reporting errors. Psychological safety, where individuals can speak up without fear, punishment or humiliation, is a key prerequisite for effective communication in high-stress environments such as the operating theatre. [33]. Any interventions aimed at improving communication in the operating theatre should consider the psychological well-being of both trainees and senior staff before and during implementation. However, this area remains understudied and represents an important direction for future research [34].

Several limitations were identified in this study. The ORMAQ questionnaire was shortened and adapted for use in this cohort to reduce survey fatigue, which may have implications for its wider validity. The sample size was relatively small and restricted to Irish hospitals, which may restrict the generalisability of the findings internationally. Another limitation is potential response bias, as the study captured only the perspectives of anaesthesiology trainees. This may have influenced the reported communication quality of different professions (e.g. higher ratings for communication quality of the anaesthesia team over surgeons or nurses). Ideally, future research should assess perspective of all operating team members to provide a more comprehensive understanding of communication dynamics, as well as evaluate specific educational interventions aimed to improve communication.

## Conclusion

This study demonstrates the critical role of teamwork and high-quality communication in the operating theatre, from the perspective of Irish anaesthesiology trainees. The results emphasise the importance of open, safe, and effective communication between junior and senior staff, particularly between anaesthetists and surgeons. Discrepancies between best practice and current hospital communication strategies highlight the need for systemic reform to align hospital practices with trainee expectations.

Addressing these communication challenges requires hospital-wide interventions, including structured communication training, leadership development, peer-led initiatives and measures to enhance psychological safety. Implementing these changes could improve teamwork, reduce medical errors, and ultimately enhance patient safety in Ireland.

## Appendix 1

**Table:**

	*Q1*	*Q2*	*Q3*	*Q4*	*Q5*	*Q6*	*Q7*	*Q8*	*Q9*	*Q10A*	*Q10B*	*Q11*	*Q12*	*Q13*	*Q14*
Mean	3.864	4.743	3.356	3.054	2.986	1.391	2.890	3.972	1.824	4.527	2.756	1.567	3.094	1.797	2.013
SE	0.114	0.054	0.107	0.115	0.109	0.097	0.114	0.103	0.104	0.067	0.121	0.077	0.126	0.096	0.082
Median	4	5	4	3	3	1	3	4	2	5	3	1	3	2	2
Mode	4	5	4	3	4	1	2	4	2	5	2	1	3	2	2
SD	0.983	0.469	0.918	0.991	0.943	0.841	0.979	0.890	0.896	0.578	1.044	0.663	1.087	0.827	0.711
	*Q15A*	*Q15B*	*Q16*	*Q17*	*Q18*	*Q19*	*Q20*	*Q21*	*Q22*	*Q23*	*Q24*	*Q25*	*Q26*	*Q27*	*Q28*
Mean	4.108	2.229	4.270	1.945	2.648	4.391	3.547	3.283	3.513	2.081	3.945	1.808	2.534	3.82	3.743
SE	0.089	0.125	0.096	0.090	0.120	0.081	0.099	0.114	0.114	0.091	0.094	0.066	0.115	0.08	0.117
Median	4	2	4	2	2	4	4	3	4	2	4	2	2	4	4
Mode	4	2	4	2	2	5	4	4	4	2	4	2	2	4	4
SD	0.768	1.079	0.832	0.774	1.039	0.698	0.850	0.986	0.982	0.789	0.809	0.569	0.987	0.746	1.007
